# APPLICATION OF ANTERIOR MINI-INCISION VERTEBROPLASTY IN CERVICAL METASTASES

**DOI:** 10.1590/1413-785220253302e288709

**Published:** 2025-10-13

**Authors:** GUAN SHI, HAO CHEN, PU JIA, LI BAO, FEI FENG

**Affiliations:** 1. Capital Medical University, Beijing Friendship Hospital, Department of Orthopaedics, Beijing, China.

**Keywords:** Neoplasm Metastasis, Vertebroplasty, Cervical Vertebrae, Metástase Neoplásica, Vertebroplastia, Vértebras Cervicais

## Abstract

**Objective:**

To investigate the efficacy and safety of anterior cervical mini-incision vertebroplasty in the treatment of vertebral metastases.

**Methods:**

From July 2009 to March 2013, seven cases of vertebral metastases were treated by using vertebroplasty through an anterior cervical paratracheal mini-incision guided by C-arm X-ray in Beijing friendship hospital, Capital medical university. Among them, three were male and four were female, aged 51 to 74 years with an average age of 61.7 years. Preoperative and postoperative Visual Analog Scale (VAS) scores and analgesic medication usage were evaluated, and postoperative pain relief was assessed using the World Health Organization (WHO) criteria.

**Results:**

All seven surgeries were successful without any occurrences of complications such as nerve or vascular injury, pulmonary embolism, or hematoma. The average cement injection volume was 1.8ml, and postoperative X-rays and CT scans indicated satisfactory cement filling, with two cases showing paravertebral cement leakage, but without clinical symptoms. One week postoperatively, the VAS score decreased from a preoperative average of 8.86 to 2.14, with complete pain relief in three cases, leading to the cessation of analgesic drugs, and partial pain relief in four cases, resulting in a reduction or downgrade of analgesic medications. Follow-ups ranged from 3 to 28 months, with one patient dying at 3 months postoperatively, one at 4 months, two at 6 months, one at 8 months, and one at 17 months, while one patient survived 28 months postoperatively. Postoperatively, all patients showed no worsening of local pain symptoms, and the surgical efficacy remained stable.

**Conclusion:**

Anterior cervical mini-incision vertebroplasty is a precise and effective method for pain relief in the treatment of vertebral metastases, providing a safe approach that reduces the risk of damaging critical cervical tissues during the puncture procedure and postoperative hematoma formation. Level of Evidence lll; Retrospective Study.

## RESUMO

**Objetivo:**

Investigar a eficácia e a segurança da vertebroplastia por mini-incisão anterior cervical no tratamento de metástases vertebrais.

**Métodos:**

Entre julho de 2009 e março de 2013, sete casos de metástases vertebrais foram tratados com vertebroplastia utilizando uma mini-incisão paratraqueal anterior cervical guiada por fluoroscopia (raios X com intensificador de imagem – C-arm) no Hospital da Amizade de Pequim, Universidade Médica Capital. Dentre os pacientes, três eram do sexo masculino e quatro do sexo feminino, com idades entre 51 e 74 anos (média de 61,7 anos). Avaliaram-se os escores da Escala Visual Analógica (EVA) e o uso de analgésicos no pré e pós-operatório, sendo o alívio da dor classificado de acordo com os critérios da Organização Mundial da Saúde (OMS).

**Resultados:**

Todas as sete cirurgias foram realizadas com sucesso, sem ocorrência de complicações como lesões nervosas ou vasculares, embolia pulmonar ou hematoma. O volume médio de cimento injetado foi de 1,8 ml. Radiografias e tomografias pós-operatórias mostraram preenchimento satisfatório do cimento, com extravasamento paravertebral em dois casos, porém sem sintomas clínicos. Após uma semana, o escore EVA médio caiu de 8,86 (pré-operatório) para 2,14, com alívio completo da dor em três pacientes (cessação do uso de analgésicos) e alívio parcial em quatro pacientes (redução ou substituição da medicação analgésica). O tempo de seguimento variou de 3 a 28 meses: um paciente faleceu com 3 meses de pós-operatório, um com 4 meses, dois com 6 meses, um com 8 meses e um com 17 meses; um paciente sobreviveu por 28 meses. Nenhum paciente apresentou agravamento dos sintomas locais de dor, e a eficácia cirúrgica manteve-se estável ao longo do tempo.

**Conclusão:**

A vertebroplastia por mini-incisão anterior cervical é um método preciso e eficaz para o alívio da dor no tratamento de metástases vertebrais, oferecendo uma abordagem segura que reduz o risco de lesão aos tecidos cervicais críticos durante o procedimento de punção e a formação de hematomas no pós-operatório. Nível de Evidência III; Estudo retrospectivo.

## INTRODUCTION

Vertebroplasty, as a minimally invasive technique for rapidly stabilizing pathological vertebrae and treating refractory pain caused by vertebral lesions, has been widely used in the treatment of vertebral hemangioma, malignant tumors, osteoporotic vertebral compression fractures, and multiple myeloma.^1,2^ Although the first reported literature on vertebroplasty was for the treatment of vertebral lesions,^3^ the low incidence rate, surgical difficulty, and high risk of vertebral lesions have limited the development of vertebroplasty for the treatment of vertebral lesions. Currently, there are relatively few domestic and international literature reports on vertebroplasty for the treatment of vertebral lesions, and most of them are presented as individual case reports.^4,5^ In this study, the author employed an anterior cervical paratracheal mini-incision, guided by a C-arm X-ray machine, to perform vertebroplasty in seven cases of vertebral metastases, aiming to explore the feasibility and clinical efficacy of anterior cervical mini-incision vertebroplasty for the treatment of vertebral metastases.

## MATERIALS AND METHODS

### Patient data

From July 2009 to March 2013, a total of seven cases of vertebral metastases were treated using anterior cervical mini-incision vertebroplasty in Beijing friendship hospital, Capital medical university, including three males and four females, with ages ranging from 51 to 74 years and an average age of 61.7 years. All patients had a confirmed primary malignant tumor, with two cases of lung cancer, two cases of breast cancer, one case of renal cancer, one case of liver cancer, and one case of colon cancer (Table 1).

**Table 1:** Baseline and follow-up of the cases.

Case	Age (years)	Primary tumor	Preoperative VAS	Postoperative VAS at 1 week	Postoperative VAS at 1 month	Postoperative VAS at 3 months	Follow-up
1	51	Lung	10	3	3	3	Died in April
2	74	Colon	8	1	1	1	Died in June
3	63	Liver	8	2	2	2	Died in September
4	56	Lung	9	1	1	0	Died in July
5	56	Breast	9	1	0	0	Alive
6	64	Breast	9	4	4	4	Died in the 17th month
7	68	Kidney	9	3	4	Died	

### Clinical assessment

All seven patients had significantly restricted neck movements and experienced severe pain with slight neck movements. Neck rotation (left and right) was limited to within 20°, backward movement was 0°, and forward bending was approximately 15°-20°. Six patients required a neck brace preoperatively to control pain. Pain severity was graded using the Visual Analog Scale (VAS):^6^ Grade 0 (0 points, no pain), Grade 1 (less than 3 points, mild pain), Grade 2 (4-6 points, moderate pain), Grade 3 (7-10 points, severe pain). All seven patients experienced severe pain (Grade 3) in the cervical vertebral region and required narcotic analgesics.

### Preoperative preparation

**Figure 1:**
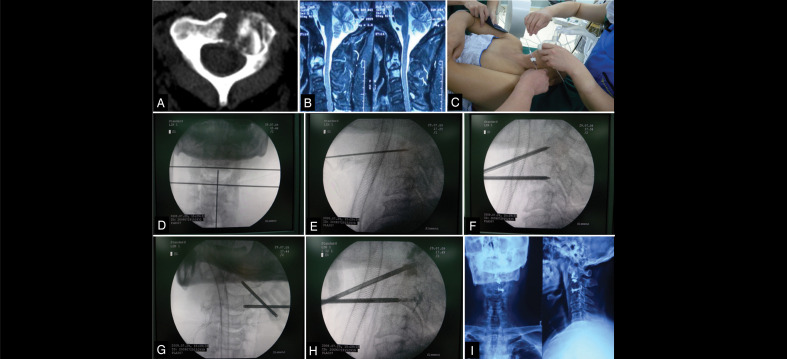
Preoperative CT showing lytic destruction of the vertebral body. B: Preoperative MRI demonstrating tumor invasion and signal changes in C2 and C3 vertebral bodies. C and D: Surface localization of the pedicle awl, determining the incision site. E-H: Fluoroscopic-guided simultaneous positioning, puncture, and bone cement infusion of the pedicle awl at C2 and C3. I: Postoperative X-ray showing satisfactory distribution of bone cement.

Source: Authors.

Routine preoperative examinations were conducted, including blood, urine, and stool tests, biochemical tests, blood gas analysis, coagulation function tests, and electrocardiograms. Cervical X-rays, CT, and MRI scans were performed to assess the extent of vertebral destruction. CT revealed osteolytic destruction of the vertebrae (Figure 1a), and MRI showed vertebral erosion and signal changes due to the tumor invasion (Figure 1b).

### Surgical procedure

The specific surgical steps were as follows. The incision site was determined on the left or right side of the trachea at the level of the mandibular angle (Figure 1c and 1d). After endotracheal intubation under general anesthesia, the patient was placed in a supine position with a cushion under the shoulders. Routine disinfection and draping were performed, and a horizontal incision of approximately 2 cm was made through the skin. The surgeon palpated the anterior cervical fascia and the anterior edge of the vertebra. The Jamshidi needle was fixed approximately one-third of its length to the anterior edge of the vertebra, and its position was confirmed under X-ray fluoroscopy (Figure 1e). The position was confirmed again under X-ray fluoroscopy in both anterior-posterior and lateral views (Figure 1f and 1g). Once the position was deemed satisfactory, bone cement was prepared. The bone cement was injected into the vertebral body under fluoroscopic guidance during the dough phase (transitioning from a stringy to a toothpaste-like consistency, Figure 1h). The puncture site was compressed manually for 3-5 minutes after the needle was removed to prevent bleeding and the formation of hematomas. The incision was closed layer by layer, completing the surgery.

### Treatment evaluation

On the second day after surgery, X-ray and CT examinations were performed to observe the distribution of the cement.VAS was reassessed one week after surgery, at one month and three months after the procedure. The postoperative pain relief was evaluated using the WHO standard^7^ at one week after surgery, as follows: complete relief, partial relief, mild relief, ineffective. The effective treatment included complete relief + partial relief, while ineffective treatment included mild relief + no improvement.

### Declarations

The requirement of ethical approval for this was waived by the ethic committee of Beijing Friendship Hospital, Capital Medical University. The need for written informed consent was waived by the ethic committee of Beijing Friendship Hospital, Capital Medical University due to retrospective nature of the study.

## RESULTS

All seven cases in this group had successful surgeries, with an average operation time of 53 minutes and an average cement injection volume of 1.8ml. All wounds healed as Class I/A, and no complications such as nerve or vascular injury, pulmonary embolism, or hematoma occurred. Follow-up X-rays and CT scans on the second day after surgery showed satisfactory cement filling in all patients (Figure 1i), with two cases showing cement leakage into the paravertebral soft tissue, but without clinical symptoms.

### Treatment efficacy

All seven patients experienced varying degrees of soft tissue swelling around the neck incision postoperatively, with no bleeding or hematoma formation along the puncture route. Swallowing movements caused discomfort, but with the use of anti-inflammatory and analgesic drugs, all patients could tolerate the symptoms, and they largely disappeared one week after surgery. The average VAS score one week after surgery decreased to 2.14, and according to the WHO standard, three cases achieved complete relief, and four cases achieved partial relief, resulting in an overall effective rate of 100%. After one week of orthopedic discharge, patients continued with systemic tumor treatment in other departments. All seven patients were followed up, with one patient dying at three months after surgery, one at four months, two at six months, one at eight months, one at seventeen months, and one patient surviving 28 months after surgery. During the follow-up period, there was no aggravation of local pain symptoms, and the surgical efficacy remained stable. VAS scores at different time points (preoperative, one week, one month, and three months after surgery) are shown in Table 1.

## DISCUSSION

For patients with advanced tumors and bone metastases, the primary goal of orthopedic surgeons is to improve the quality of life and alleviate the pain. The traditional surgical treatment for cervical spine metastases involves tumor resection, decompression, and vertebral body reconstruction. However, this procedure is associated with significant trauma, high bleeding risk, and high cost. Conservative treatments include local immobilization, wearing a cervical collar, and oral analgesics, but the efficacy is often unsatisfactory. Radiation therapy can relieve pain to some extent,^8^ but it cannot enhance the stability of the affected vertebrae. In cases of pathological fractures, instability of the atlantoaxial structure can lead to damage to the upper cervical cord, nerve roots, or blood vessels, resulting in high cervical myelopathy.

For the treatment of spinal bone metastases, vertebroplasty has advantages such as minimal invasiveness, low risk, rapid pain relief, and fast patient recovery. It can stabilize the vertebral body. However, due to the complex distribution of nerves, blood vessels, trachea, and esophagus, vertebroplasty for cervical spine lesions is more challenging. In this study, vertebroplasty for cervical spine were successfully operated to all seven patients.

### Surgical method of vertebroplasty for cervical spine metastases

The transoral puncture approach requires complex preoperative preparation, including consultation with the oral and maxillofacial department to assist with oral cleaning. Intraoperatively, general anesthesia and tracheal intubation are needed, along with the use of a joint retractor, and cooperation among multiple departments, including oral and maxillofacial surgery. This approach carries a relatively higher risk of postoperative bleeding and infection, with an infection rate as high as 6.5%.^9,10^ Patients may experience discomfort in the throat postoperatively, leading to limited oral intake, necessitating symptomatic treatment such as humidifying and inhaling sputum-dissolving agents.

The anterolateral approach is a classic approach for vertebroplasty in the treatment of cervical spine lesions, and its safety and efficacy have been recognized.^11^ However, due to the high position of the atlantoaxial joint, the anterolateral approach requires the patient’s head to be excessively tilted backward, lifting the mandible as much as possible to avoid interference with the puncture. This positioning often leads to increased pain, making it difficult for patients to cooperate, sometimes requiring surgery under general anesthesia. The atlantoaxial joint is surrounded by many vital structures, such as the accessory nerve, hypoglossal nerve, vagus nerve, carotid artery, and thyroid artery and vein.^12^ The percutaneous puncture approach minimizes damage but is difficult to perform in obese patients. Therefore, to reduce the risk of injury to nerves and blood vessels during the puncture process, a modified anterolateral approach was used in this study. After general anesthesia, a transverse incision was made near the esophagus, and the subcutaneous tissue, fascia, and muscle layers were cut open. The sternocleidomastoid muscle was accessed along its inner edge, and the esophageal and vascular sheaths were bluntly separated to protect and pull the trachea and esophagus, minimizing traction on the carotid sheath to prevent cardiovascular reactions caused by vagus nerve or carotid sinus traction. The surgeon’s fingers directly touched the anterior edge of the atlantoaxial joint, and then the puncture was performed, avoiding important anterior structures. The blood supply to the surface of the atlantoaxial joint is abundant, and after the injection of bone cement, the puncture site was compressed with fingers for 3-5 minutes to prevent bone surface and muscle bleeding.

### Preventive measures for bone cement leakage

Bone cement leakage is the most common complication of vertebroplasty, accounting for more than 50% of all clinical complications.^13^ Bone cement leakage can occur in various locations, such as extradural leakage, foraminal leakage, disc leakage, paraspinal soft tissue leakage, paravertebral venous leakage, and puncture needle tract leakage. The vast majority of bone cement leakages do not cause clinical symptoms. A meta-analysis by Eck et al.^14^ including 3,034 patients who underwent bone cement surgery, found that the overall incidence of bone cement leakage was 7%, while the incidence of symptomatic leakage was 0.3%. The vertebrae with metastases often have rich venous plexuses. During bone cement injection, the anesthesiologist should be vigilant and monitor the patient’s vital signs to guard against the occurrence of pulmonary embolism. While a small amount of PMMA entering the vertebral canal and pulmonary artery does not cause clinical symptoms, once a certain amount is reached, it can lead to serious consequences. Patients may experience symptoms such as dyspnea, tachycardia, decreased blood oxygen levels, coughing, and sputum production, and even death.^15^

Leakage of bone cement into the spinal canal or intervertebral foramen at the level of the atlantoaxial joint can cause compression of the spinal cord or nerve roots, leading to radicular symptoms or high cervical myelopathy, resulting in catastrophic consequences. The author suggests that patients with this condition should undergo comprehensive X-ray, CT, and MRI examinations before surgery. During the puncture procedure, the needle tip should be positioned as far away from the endplates as possible. The instrument nurse should strictly follow the proportion to prepare the bone cement. When it reaches a slurry-like consistency, it is placed into the spiral pressurization device for approximately 1 minute. A small amount of bone cement is then extruded to observe its transition from a stringy state to a toothpaste-like state. Subsequently, the injection is performed under fluoroscopic guidance. The injection process should be slow to reduce the injection pressure. Alternatively, 0.5 ml can be injected first, and after waiting for about 15-20 seconds for the cement to disperse and stabilize within the vertebra, the distribution of bone cement is observed before continuing the injection. As soon as bone cement reaches the posterior wall of the vertebra or the paravertebral venous plexus is visualized, the injection should be immediately stopped. It has been reported^16^ that the filling volume of bone cement in the lesion area is not directly proportional to the analgesic effect. Therefore, one should not intentionally pursue excessive bone cement filling, which could increase the risk of cement leakage.

The clinical efficacy of vertebroplasty in treating atlantoaxial metastases has been validated. In our opinion, patients without indications for resection of atlantoaxial metastases can undergo vertebroplasty to reinforce the stability of the affected atlantoaxial joint. We have successfully improved the classic anterolateral approach and achieved favorable clinical results, effectively reducing the risk of injury to vital structures in the anterior neck, with a high success rate and practical clinical value.
